# Association between problematic Internet use and specific Internet activities and COVID-19- and earthquake-related stress, anxiety, and depression symptoms among Croatian young adults

**DOI:** 10.3389/fpsyt.2023.1227182

**Published:** 2023-08-09

**Authors:** Zrnka Kovačić Petrović, Tina Peraica, Mirta Blažev, Dragica Kozarić-Kovačić

**Affiliations:** ^1^Department of Psychiatry and Psychological Medicine, University of Zagreb, School of Medicine, Zagreb, Croatia; ^2^Department of Addiction, University Psychiatric Hospital Vrapče, Zagreb, Croatia; ^3^Department of Psychiatry, Referral Center for Stress-related Disorders of the Ministry of Health, University Hospital Dubrava, Zagreb, Croatia; ^4^Department of Forensic Sciences, University of Split, Split, Croatia; ^5^Ivo Pilar Institute of Social Sciences, Zagreb, Croatia

**Keywords:** young adults, Internet use, COVID-19, earthquakes, mental health

## Abstract

**Background:**

During the COVID-19 pandemic and concomitant earthquakes in Croatia in 2020, increased Internet use (IU) and Internet-based addictive behaviors were associated with decreasing mental well-being. We determined the changes in IU, problematic IU (PIU), and problematic specific Internet activities in young adults during the prolonged stress caused by the pandemic and earthquakes, age differences in PIU and differences in perceived source of stress (pandemic or earthquakes), and association between PIU and increase in specific Internet activities and stress, anxiety, and depression symptoms in young adults.

**Methods:**

A cross-sectional online survey conducted from September 30, 2021 to October 17, 2021 included 353 young adults aged 22.6 ± 2.1 years, 382 early adults aged 32.1 ± 4.4 years, and 371 middle-aged adults aged 49.0 ± 6.5 years. Data on sociodemographic characteristics, stressors (without perceived stressors, only pandemic-related stressor, only earthquake-related stressor, and both pandemic and earthquake-related stressors), PIU and IU were collected with a self-report questionnaire. The Impact of Event Scale and the Hospital Anxiety Depression Scale were used to evaluate mental symptoms. PIU and problematic specific Internet activities were assessed using Tao et al.’s criteria. Data were anaylzed with paired-sample Wilcoxon test, McNemar’s and Pearson’s chi-square tests, and structural equation modeling.

**Results:**

In 17% of young adults, we found increased PIU (OR = 5.15, 95% CI [2.82, 10.18]), problematic social media use (OR = 2.77, 95% CI [1.56, 5.14]), and uncontrolled online shopping (OR = 5.75, 95% CI [1.97, 22.87]) (*p* < 0.001 for all). PIU and problematic social media use were more common among young adults (60.8%), as well as problematic online gaming (25.9%). Problematic social media use was more frequent among young adults reporting pandemic stress than among those without perceived stress (69.9% vs. 43.2%). Increased online gaming predicted more severe avoidance symptoms (*p* = 0.041), increased social media use predicted more severe depression symptoms (*p* = 0.017), increased online shopping predicted more severe intrusion (*p* = 0.013) and anxiety symptoms (*p* = 0.001). PIU predicted more severe intrusion (*p* = 0.008), avoidance (*p* = 0.01), anxiety (*p* < 0.001), and depression (*p* = 0.012) symptoms.

**Conclusion:**

Different effects of the pandemic and earthquakes on IU could reflect a different effect of various stressors on Internet behavior of young adults. Type of problematic Internet behavior may predict for the type of mental health problem.

## Introduction

In the recent years, problematic Internet use (PIU) has multiplied due to the growing popularity of the Internet, especially among adolescents and young adults. They are at high risk for PIU due to their immature ability to exert self-control (1) and availability of free time (2). Young adults’ cognitive and self-regulatory skills may increase gradually over time (3), and their leisure time may decrease as academic tasks increase.

Internet addiction (IA), a concept first used in the late 1990s, reflects a conceptual similarity to addiction to psychoactive substances. It is an umbrella term that refers to a set of online behaviors (gambling, online gaming, pornography viewing, social media, online shopping, etc.) that can become addictive (4). Many authors criticize the concept of IA because of the “Internet” component and the “addiction” component. Several authors proposed the use of concepts referring to specific online activities instead IA. However, research activities led to the inclusion of gambling and gaming disorders in the eleventh edition of the International Classification of Diseases (5) as disorders caused by addictive behavior. Various alternative terms have been proposed to address the above criticisms, such as problematic Internet use (PIU), excessive Internet use, pathological Internet use, compulsive Internet use, and Internet use disorder. Similar terms are used for specific Internet activities (e.g., problematic gaming, problematic gambling, compulsive social media use, uncontrolled or compulsive shopping, etc.). PIU is an umbrella term for the pathological level of specific Internet activities, including excessive social media use, gaming, gambling, streaming, pornography viewing, impulsive buying, and newer behaviors such as cyberhoarding and cyberchondria (4, 6).

The key developmental tasks of young adults aged between 18 and 25 are identity formation and self-exploration (7). This neurobio-psychologically specific developmental period (8) differs from adolescence on one hand and adulthood on the other. However, there is no single definition of young adulthood and the age range for this developmental period, which may be a source of confusion in research and prevention and treatment programs (7).

The normal development of young adults can be disrupted by traumatic events that can influence PIU (9) or alter the tendency to experience PIU (10). Traumatic events, such as natural disasters, abuse, traffic accidents, or loss of loved ones can be an important external stimulus for PIU (11, 12), may elicit different negative psychological responses and have different psychological consequences (13–16) if experienced at this age and especially in young people with lower mental health literacy (17, 18). Exposure to trauma also has negative effects on their behavior, social, and emotional functioning as well on their academic achievements (19).

There are several hypotheses about negative reactions to traumatic events and experiences in sensitive developmental periods, such as adolescence or young adulthood, although the connection is unclear. According to the self-determination theory, if the basic psychological needs including autonomy, competence, and relatedness are not satisfied, negative behaviors can ensue, including excessive use of the Internet (20). According to the loss-of-compensation theory, adolescents and young people can compensate for unsatisfied basic psychological needs by excessively using the Internet, which can lead to Internet addiction (IA) (21). PIU can reduce the suffering of individuals who have faced traumatic experiences and therefore PIU can be considered a dysfunctional coping strategy for trauma (22).

The appearance of mental symptoms (stress, anxiety, and depression) may indicate that these needs are not met and, in case of stressful experiences due to severe adversity (23), adolescents and young adults may turn to the Internet for psychological satisfaction.

The situation in Croatia was specific because two devastating earthquakes occurred during the COVID-19 pandemic, affecting a large part of the Croatian population. The first earthquake hit Zagreb (the capital city) on March 22, 2020, during the first three waves of COVID-19 outbreak (first: from mid-March to early May 2020; second: from late September to mid-February 2021; third: from mid-February to early June 2021) (24). The other hit the town of Petrinja (about 80 km SE from Zagreb) on December 29, 2020, and it was felt as far as Zagreb.

There is ample evidence of increased Internet use during the COVID-19 pandemic, including Internet-based addictive behaviors (25). Furthermore, there is accumulating evidence on the relationship between COVID-19 pandemic and quality of life, mental health, risk factors, and vulnerable groups (25, 26).

In the last two decades, the use of the Internet has significantly increased (27), especially between 2020 and 2022 (28). The effects were beneficial when the Internet was used for work, school, learning, social communication, entertainment, stress relief, and psychotherapy; however, uncontrolled Internet overuse could also lead to PIU/IA or problematic specific Internet activities (29, 30). In comparison with the prepandemic period, the prevalence rates of PIU and specific Internet activities and time spent on the Internet during the pandemic were higher in adults (31–34), adolescents (26, 35, 36), younger generation (33, 34, 36), and males (26).

Due to the impact of the COVID-19 pandemic on mental health, it was assumed that the ability to gamble directly from home would increase the rate of problem gambling (25). A review articles found that, contrary to the expectations, gambling behavior often decreased or remained the same for most gamblers during the pandemic, and the only increase in gambling behavior was associated with problem gambling (37, 38). Problem gambling among gacha gamers in Chinese young adults was associated with greater stress and higher anxiety level, with higher proportion of female participants in high-risk group of becoming problem gamblers (39).

A significant increase in online gaming was observed during the COVID-19 stay-at-home order (40). Initiatives, such as #PlayApartTogether, which promote gaming for socializing and stress reduction could have positive results during the pandemic if approaches to gaming were balanced and effective (40). Internet gaming disorder increased in the Japanese adults, especially among younger persons (34), and online gaming increased among Indian college students (41) and Malaysian medical students (42). One systematic review found that, although video games, particularly augmented reality and online multiplayer games, alleviated stress, anxiety, depression and loneliness among adolescents and young adults during stay-at-home restrictions, in at-risk individuals (particularly male youths), video games had a harmful effect (43).

Digital and communication technology has provided useful communication during the pandemic and natural disasters, especially for the maintenance and development of interpersonal relationships and for education and work. Due to the significantly increased use of social networks, adverse psychological and behavioral effects may occur (25). For example, social media addiction negatively affected Polish women’s (mean age 23.07 ± 4.69) sexual functioning during the COVID-19 pandemic (44). One systematic review and meta-analysis found negative relationship between mobile phone addiction and physical activity among young adults (45).

Increased pornography use was found among adults (46), especially during the quarantine (33), while the use of pornography among adolescents was rather stable between November 2019 and June 2021, i.e., during the COVID-19 pandemic (47).

Although online shopping is the safest way to prevent exposure to the virus, it can turn into uncontrolled shopping and can harm mental health. One US study found that the most popular activities during the pandemic for adults were gaming and uncontrolled online buying (48). Another study reported that uncontrolled online shopping and web navigation may be modulators of unpleasant emotional states (49).

In times of crisis, such as pandemics and natural disasters, children and adolescents are particularly vulnerable to develop mental disorders (50). The main potential risk factors for the development of PIU are age (children, adolescents, young adults), male gender, depression, anxiety, and stress (50–52). Anxiety, depression, stress, and impulsivity in frequent Internet users were identified as risk factors for PIU/IA (53–55).

An Italian study of the consequence of the earthquake among L’Aquila students reported 30% of PIU, with older students being more likely to develop a PIU irrespective of gender and educational level (56). Early detection of such behavior among youth is important in order to structure interventions to prevent abnormal functioning (56). A Chinese study found that post-traumatic stress symptoms (PTSS) (cognitive symptoms, negative mood alterations, intrusion and hyperarousal symptoms) in adolescents exposed to a major earthquake had positive predictive effects on IA, while positive beliefs about adversity did not have buffering effects on the relationship between the PTSS symptoms and IA (57). Another Chinese follow-up study after the Wenchuan earthquake found that PIU in adolescents did not remain stable, but showed heterogeneous trajectories over time. Age, gender, and post-trauma fear rather than trauma exposure itself may serve as predictors for distinguishing PIU trajectories (58).

PIU or specific Internet activities during the COVID-19 pandemic or earthquakes among young adults (39, 59–61), adolescents (57, 58) and students (41, 53, 57, 62, 63) have already piqued researchers’ interest. The transition to adulthood can be a vulnerable period, particularly in young adults aged 18–25 years who experienced traumatic events. The first rationale of this study was that pandemic- and earthquake-related stress can be the triggers for PIU and problematic specific Internet activities. The second rationale was that stress, anxiety, and depression symptoms are associated with PIU and specific Internet activities, as found in the previous research. Previous research studies investigating the effects of pandemic- and earthquake-related stress on PIU and specific Internet activities mainly focused on adolescents and students, i.e., subgroups of young adults, but the age ranges differed due to various definitions of young adulthood. This is the first study that explored the effects of two concurrent stressful events (pandemic and earthquakes) on Internet based addictive behaviors and mental health in the exposed young adults.

Our study had five aims. The first one was to check for the increase in daily overall Internet use and specific Internet activities (online gaming, online gambling, online shopping, social media use, and pornography viewing) in young adults during the prolonged stress caused by COVID-19 pandemic and earthquakes. The second one was to compare the overall PIU and problematic specific Internet activities in young adults before vs. during the pandemic and earthquakes period. The third aim was to examine the differences in PIU and problematic specific Internet activities between young adults (18–25 years), early adults (25–40 years), and middle-aged adults (40–65 years). The fourth aim was to determine the differences between the experienced and perceived stress in young adults in relation to PIU and problematic specific Internet activities. The last aim was to apply a structural equation modeling to test the hypothesized model, examining the association between the increase in PIU and specific Internet activities during a prolonged stress period, and the elevated levels of stress, anxiety, and depression symptoms in young adults.

## Participants and methods

### Study design

The hyperlink to a 20-min online survey created by authors was sent to the eligible recipients via social network platforms including LinkedIn, Facebook, Google+, Twitter, and Instagram. Before taking the survey, which was made available on a Google-built website, the participants could read the description of study aims and participants’ rights on the first page of the survey.

The data were self-reported and collected from September 30, 2021 to October 17, 2021.

As this study was part of a larger research project, the dataset was the same as the one used in a previous study (64). Only the methods relevant for the present analysis are described here, while a detailed description may be found in the earlier published article (64).

### Participants

Of 1,286 people who completed the online survey, 146 submitted incomplete questionnaires and 22 were minors and were excluded from the analysis (*n* = 168). The final sample consisted of 1,118 individuals over 18 years of age (220 men and 898 women, mean age 35.1 ± 12.3 years; age range 18–78 years). Participation was voluntary and no financial compensation was offered. Inclusion criteria were being a Croatian resident and older than 18 years. The exclusion criteria were as follows: not understanding written Croatian language, not signing the informed consent, and missing data. Informed consent was given by all participants in accordance with the international ethical principles in human research.

We divided our initial study sample of 1,118 participants in four age groups: young adults (18–25 years), early adults (25–40 years), middle-aged adults (40–65 years), and older adults (>65 years). The group of older adults was not included in further analysis due to a small sample size (*n* = 12). Of 1,106 analysed participants, 353 were young adults, 382 were early adults and 371 were middle-aged adults.

### Ethical considerations

All participants included in the study provided informed consent in accordance with the ethical principles in human research. Participation was voluntary and excluded financial or any other compensation. The Ethics Committee of the University Hospital Vrapče, in Zagreb, Croatia approved the study (Prot. 23-1064/3-21).

### Measures

*Sociodemographic data* (age, gender, education level, occupational, marital, and parental statuses) and *data on the Internet use* were collected.

*Increase in overall Internet use and specific Internet activities* was measured using a dichotomous (no/yes) question: “Have you increased your overall and specific Internet activities use (online gambling, online gaming, social media, online shopping, pornography viewing) during the prolonged stress (COVID-19 pandemic and earthquakes)?,” separately for overall Internet use and each specific Internet activity. The total score was either 0 – no increase or 1 – increase.

*PIU and problematic specific Internet activities* were measured using a modified, 8-item symptom list, according to the criteria proposed by Tao et al. (65). PIU and problematic specific Internet activities were considered present if the participants checked two symptoms: (1) preoccupation and (2) withdrawal symptoms plus any of the other symptoms, which included (3) tolerance; (4) lack of control; (5) continued excessive use of the Internet despite being aware of negative effects/affects; (6) loss of other interests; (7) using Internet to avoid or alleviate dysphoric mood; and (8) withdrawing from friends, relatives or important relationship or missing career opportunities because of Internet use, and duration of Internet use (6 h or more daily for at least 3 months) before the first pandemic wave in March 2020 and during the first three pandemic waves and concurrent earthquakes. Participants could select yes or no answers for PIU and each of the problematic specific Internet activities. The total scores were dichotomized, with 0 indicating absence and 1 indicating presence of PIU and problematic specific Internet activities. Kuder Richardson-20 (66) internal reliability was 0.85 for PIU (0.84 young adults; 0.86 early adults; 0.85 middle-aged adults), 0.80 for online gaming (0.81 young adults; 0.80 early adults; 0.79 middle-aged adults), 0.80 for pornography viewing, 0.82 for social media use (0.84 young adults; 0.80 early adults; 0.82 middle-aged adults), and 0.81 for online shopping (0.83 young adults; 0.81 early adults; 0.81 middle-aged adults).

To identify *the source of experienced stress*, three *ad-hoc* developed questions were used. Participants were asked if they perceived COVID-19 pandemic as stressful (no/yes) and to indicate how stressful the Zagreb and Petrinja earthquakes were for them on a 5-point Likert scale (1 = not at all, 2 = low stressful, 3 = moderate stressful, 4 = stressful, 5 = very stressful). On the basis of self-reported stress, participants were divided into four groups: no perceived stress, only pandemic-related stress, only earthquake-related stress, and both pandemic- and earthquake-related stress.

*Stress symptoms* were measured with the Impact of Event Scale (IES) (67), a 15-item questionnaire, to evaluate experiences of avoidance and intrusion, which “reflect the intensity of the post-traumatic phenomena.” Participants were asked to indicate on a 4-point Likert scale (not at all = 0; rarely = 1; sometimes = 3; often = 5) the stress symptoms experienced only in the week before data collection. Cronbach’s α in our sample of young adults was 0.93 for IES, 0.92 for IES intrusion, and 0.89 for IES avoidance.

*Anxiety and depression symptoms* were measured with the Hospital Anxiety Depression Scale (HADS) (68), consisting of 14 questions detecting the symptoms of anxiety (HADSA) and depression (HADSD). Participants were asked to indicate on a 4-point Likert scale (0 = not at all, 3 = all the time) their anxiety and depression symptoms experienced only in the week before data collection. Cronbach’s α in our sample of young adults was 0.91 for total HADS score, 0.89 for HADSA subscale, and 0.83 for HADSD subscale.

### Statistical analysis

The univariate normality of distribution for all continuous variables was tested with Shapiro–Wilk’s test. Increase in overall daily Internet use and specific Internet activities during the prolonged stress period was determined using the paired samples Wilcoxon test. Effect sizes were reported with *r,* according to Cohen’s (69) criteria: 0.1–0.3 indicates small, 0.30–0.50 moderate and > 0.50 large effect size, respectively.

McNemar’s chi-square test with the continuity correction was used to assess the overall amount of PIU and problematic specific Internet activities increase during the prolonged stress period. Effect sizes were reported as odds ratio (OR) with 95% confidence interval (CI).

To determine the age differences (young, early, and middle-aged adults) and differences in the self-reported perceived stress among young adults in regards to PIU and problematic specific Internet activities, Pearson’s chi-square test (i.e., Fisher’s exact test if the expected cell count was less than 5) was used, with Bonferroni adjustment. Effect sizes were reported using Cramer’s V where, according to Cohen’s (69) criteria, values 0.1 were considered to reflect low, 0.3 medium, and 0.5 high strength of association between dichotomous variables.

Finally, structural equation modeling with weighted least square mean and variance adjusted (WLSMV) estimator that is robust when non-normality is present in the data (70, 71) and full information maximum likelihood (FIML) method for handling any missing variables was used to test the model in which PIU and increase in specific Internet activities during prolonged stressful period predicted stress, anxiety and depression symptoms among young adults. In examining model-data fit, Hu and Bentler’s (72) and Browne and Cudeck’s (73) cut-off criteria were consulted for different goodness-of-fit indices: chi-square (non-significant *p*-value indicates good fit), normed chi-square (χ^2^/df < 3 good/ <5 acceptable fit), the comparative fit index (CFI) and the Tucker-Lewis index (TLI) (CFI and TLI; >0.95 good/>0.90 acceptable fit), the root mean square error of approximation (RMSEA) and its 90% confidence interval and the standardized root mean square residual (SRMR; <0.08 good/<0.10 acceptable fit). Standardized regression coefficients (β), their *p*-values, and 95% confidence intervals (CI) for regression paths in the tested model were also reported.

All statistical analyses were performed using the The jamovi project (Version 2.3) (74).

## Results

### Sociodemographic distribution and normality assumption check

The mean (±SD) age of young adults was 22.6 ± 2.1 years. Majority were women (80%), students (58%), single (48%) or in a relationship (46%), and without children (99%). The mean age of early adults was 32.1 ± 4.4 years. Majority were women (74%), employed (83%), married (49%) or in a relationship (26%), and without children (69%). The mean age of middle-aged adults was 49.0 ± 6.5 years. Majority were women (87%), employed (82%), married (60%) or divorced (15%), with children (70%).

All tested continuous variables significantly deviated from normality (Shapiro-Wilk’s test, *p* < 0.001). Therefore, tests that do not assume normality of distribution were used in all further analyses (non-parametric paired samples Wilcoxon test, McNemar’s chi-square test, and Pearson’s chi-square test) or that are robust enough to tolerate certain departures from normality (structural equation modeling with WLSMV estimator) (70, 71).

### Determining increase in overall Internet use and specific Internet activities during the prolonged stress period among young adults

Before the pandemic and earthquakes, only 2.5% young adults gambled online, while more than a third engaged in online gaming (38%) and pornography viewing (42%) (Table 1). More than half of young adults were engaged in online shopping (65%), while almost everyone was using social media (94%) and overall Internet use (96%). During the pandemic and earthquakes period, most young adults did not increase their use regarding online gambling, online gaming, pornography viewing, and online shopping. Only 2% of young adults reported increase in online gambling, less than 15% reported increase in online gaming (14%) and pornography viewing (11%), and almost a third reported increase in online shopping (30%). More than half of the young adults reported increased social media (55%) and overall Internet use (58%).

**Table 1:** Increase in overall Internet use and specific Internet activities during the pandemic and earthquakes among young adults—descriptive statistics and the paired samples Wilcoxon test (*N* = 353).

		Frequency in use		Daily use (in hours)				
		Before pandemic and earthquakes	Increase during pandemic and earthquakes	Before pandemic and earthquakes	During pandemic and earthquakes			
		*n* (%)	*n* (%)	*M* ± SD	*M* ± SD	*z*	*p*	*r*
OGB	No	344 (97.5)	346 (98.0)	1.14 ± 0.38	1.29 ± 0.76	–^a^	–^a^	–^a^
	Yes	9 (2.5)	7 (2.0)					
OG	No	220 (62.3)	302 (85.6)	1.86 ± 1.22	1.97 ± 1.29	1.01	0.311	0.11
	Yes	133 (37.7)	51 (14.4)					
PV	No	206 (58.4)	316 (89.5)	1.07 ± 0.42	1.13 ± 0.58	1.34	0.180	0.18
	Yes	147 (41.6)	37 (10.5)					
SM	No	22 (6.2)	159 (45.0)	2.80 ± 1.31	3.08 ± 1.36	4.39*	<0.001	0.25
	Yes	331 (93.8)	194 (55.0)					
OS	No	124 (35.1)	247 (70.0)	1.20 ± 0.62	1.37 ± 0.80	2.71*	0.007	0.24
	Yes	229 (64.9)	106 (30.0)					
IU	No	14 (4.0)	147 (41.6)	2.03 ± 0.97	2.46 ± 1.13	7.73*	<0.001	0.43
	Yes	339 (96.0)	206 (58.4)					

OGB, online gambling; OG, Online gaming; PV, Pornography viewing; SM, Social media; OS, Online shopping; IU, overall Internet use; a – sample size was too small (*n* = 7) for paired sample Wilcoxon test; *p < 0.01.

Self-reported daily Internet use (per hour) increased during the pandemic and earthquakes in comparison with the daily Internet use before that period (Table 1), but these differences were significant only regarding social media use (*z* = 4.39, *p* < 0.001, *r* = 0.25), online shopping (*z* = 2.71, *p* = 0.015, *r* = 0.24) and overall Internet use (*z* = 7.73, *p* < 0.001, *r* = 0.43). The results regarding social media use and online shopping indicate a small effect size, while overall daily Internet use indicates a moderate effect size.

### Differences in the overall amount of problematic Internet use and problematic specific Internet activities before and during the pandemic and earthquakes among young adults

PIU among young adults has increased during the pandemic and earthquakes (Table 2). PIU was increased in 17% of young adults, while odds of PIU were 5.15 times greater compared with the period before the prolonged stress. Regarding the specific Internet activities, 9.6% of young adults increased problematic social media use, while 4.1% increased uncontrolled online shopping during the pandemic and earthquakes. In comparison with the period before the prolonged stress, the odds for problematic social media use and uncontrolled online shopping use were 2.77 and 5.75 times greater, respectively. Problematic online gaming was not significantly increased.

**Table 2:** Differences in the overall amount of problematic Internet use and problematic specific Internet activities before and during the pandemic and earthquakes among young adults—McNemar’s chi-square test (*N* = 353).

		Problematic use					
		No	Yes				
	Internet use	*n* (%)	*n* (%)	χ^2^	*p*	OR	OR 95% CI
OGB	Before stress	7 (100.0)	0 (0.0)	–^a^	–^a^	–^a^	–^a^
	During stress	6 (85.7)	1 (14.3)				
OG	Before stress	59 (75.6)	19 (24.4)	0.07	0.789	1.33	[0.41–4.66]
	During stress	57 (73.1)	21 (26.9)				
PV	Before stress	54 (98.2)	1 (1.8)	–^a^	–^a^	–^a^	–^a^
	During stress	53 (96.4)	2 (3.6)				
SM	Before stress	150 (48.2)	161 (51.8)	13.14*	<0.001	2.77	[1.56–5.14]
	During stress	120 (38.6)	191 (61.4)				
OS	Before stress	127 (96.2)	5 (5.0)	12.00*	<0.001	5.75	[1.97–22.87]
	During stress	120 (90.9)	12 (9.1)				
IU	Before stress	254 (80.1)	63 (19.9)	35.11*	<0.001	5.15	[2.82–10.18]
	During stress	200 (63.1)	117 (36.9)				

OGB, online gambling; OG, Online gaming; PV, Pornography viewing; SM, Social media; OS, Online shopping; IU, overall Internet use; OR, odds ratio; OR 95% CI, 95% Confidence interval for odds ratio; a – cannot be computed as sample size per cell was too small for McNemar’s chi–square test; *p < 0.001.

### Differences in problematic Internet use and problematic specific Internet activities between young adults and older participants

Significant age differences were found only in problematic social media use and PIU (Table 3). At the same time, there was a marginal statistical age difference in problematic online gaming, but no significant age differences were found regarding uncontrolled online shopping. Problematic social media use and PIU were more common among young adults (60.8%) in comparison with older age groups (39.5% in early adults; 29.0% in middle-aged adults). Middle-aged adults had the lowest rate of problematic social media use (29.0%) and the same PIU as early adults (24.0% vs. 18.7%). Problematic online gaming was more frequent among young adults (25.9%) in contrast to early adults (10.3%), while no differences were found in comparison with middle-aged adults (20.0%).

**Table 3:** Differences in problematic Internet use and problematic specific Internet activities between young adults and older participants—Pearson’s chi-square test (*N* = 1,106).

	Age groups					
	Young adults	Early adults	Middle-aged adults			
Problematic use	*n* (%)	*n* (%)	*n* (%)	χ^2^	*p*	*V*
Online gambling	1 (12.5)	1 (25.0)	0 (0.0)	–^d^	–^d^	–^d^
Online gaming	21 (25.9)^a^	7 (10.3)^b^	14 (20.0)^a,b^	5.87	0.053	0.16
Pornography viewing	2 (3.6)	2 (3.9)	0 (0.0)	–^d^	–^d^	–^d^
Social media	194 (60.8)^a^	130 (39.5)^b^	93 (29.0)^c^	68.70*	<0.001	0.27
Online shopping	14 (9.5)^a^	8 (5.4)^a^	7 (6.7)^a^	1.89	0.388	0.07
Internet overall	119 (36.8)^a^	81 (24.0)^b^	62 (18.7)^b^	29.37*	<0.001	0.17

V, Cramer’s V; Different superscript letters (“a,” “b” and “c”) indicate statistically significant differences between the categories in different columns; d, cannot be computed because problematic Internet use variable is constant per specific group; *p < 0.05.

### Differences in the perceived stress among young adults in regards to problematic Internet use and problematic specific Internet activities

The only significant difference was related to different sources of stress and problematic social media use (Table 4). Specifically, young adults perceiving the pandemic as the main source of stress had more problematic social media use compared with young adults without perceived stress (69.9% vs. 43.2%). All reported differences are of low to medium effect size.

**Table 4:** Differences in the perceived stress among young adults in regard to problematic Internet use and problematic specific Internet activities—Pearson’s chi-square test (*N* = 353).

	Source of stress						
	Without stress	Pandemic	Earthquake	Pandemic and earthquake			
	*n* (%)	*n* (%)	*n* (%)	*n* (%)	χ^2^	*p*	*V*
OGB	0 (0.0)	1 (12.5)	0 (0.0)	0 (0.0)	–^c^	–^c^	–^c^
OG^d^	3 (18.8)^a^	6 (31.6)^a^	3 (33.3)^a^	9 (24.3)^a^	1.21	0.770	0.11
PV	0 (0.0)	0 (0.0)	0 (0.0)	2 (7.7)	–^c^	–^c^	–^c^
SM	19 (43.2)^a^	51 (69.9)^b^	18 (69.2)^a,b^	106 (60.2)^a,b^	9.05*	0.029	0.17
OS^d^	1 (5.0)^a^	5 (14.7)^a^	1 (7.7)^a^	7 (8.8)^a^	1.48	0.707	0.11
IU	10 (21.7)^a^	33 (44.0)^a^	9 (34.6)^a^	67 (38.1)^a^	6.33	0.097	0.14

V, Cramer’s V; Different superscript letters (“a” and “b”) indicate statistically significant differences between the categories in different columns; d – Fisher’s Exact Test; c – cannot be computed because problematic Internet use variable is constant per specific group; *p < 0.05.

### Increase in specific Internet activities use and problematic Internet use during prolonged stressful period and its association with stress, anxiety, and depression symptoms among young adults

**Figure 1:**
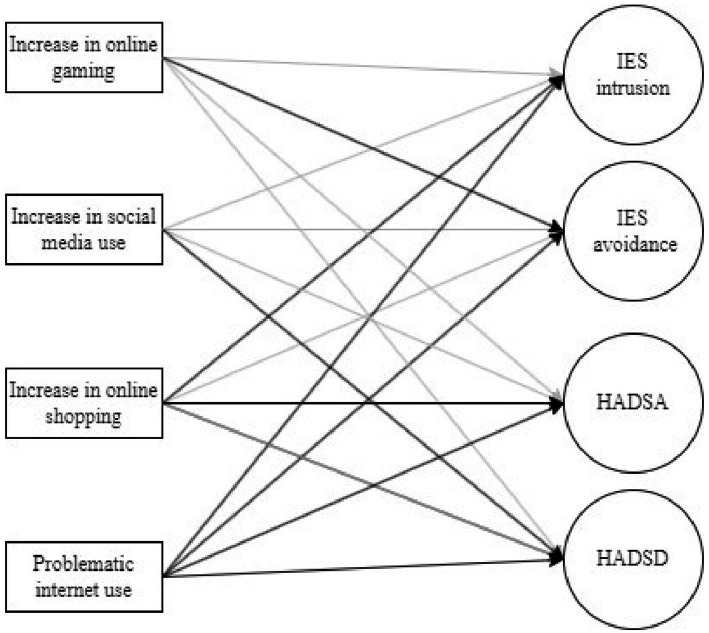
Model tested by structural equation modeling. IES intrusion, Impact of Event Scale intrusion; IES avoidance, Impact of Event Scale avoidance; HADSA, Hospital Anxiety and Depression Scale anxiety; HADSD, Hospital Anxiety and Depression Scale depression; Indicators for latent variables, as well as errors and residuals were omitted from figure due to clarity; Black arrows represents regression paths that are significant at *p* < 0.05, while gray arrows represent tested, but non-significant regression paths.

Structural equation modeling with WLSMV estimator was conducted to test the model in which increase in Internet specific activities use and PIU among young adults were defined as exogenous variables, while levels of stress, anxiety, and depression symptoms as endogenous variables (Figure 1). Results indicated that the tested model had good model-data fit on majority of goodness-of-fit indices and in accordance with Hu and Bentler’s (72) and Browne and Cudeck’s (73) cut-off criteria, i.e., χ^2^(410) = 773.02, *p* < 0.001; χ^2^/df = 1.89; CFI = 0.919; TLI = 0.910; RMSEA = 0.052, RMSEA 90% CI [0.047, 0.058], RMSEA *p*_close_ = 0.242; SRMR = 0.055.

Increase in specific Internet activities and PIU significantly explained 9.7% of intrusion, 6.8% avoidance, 17.1% of anxiety and 12.4% of depression symptoms variance among young adults (Table 5). Specifically, increase in online gaming use during the prolonged stressful period predicted more severe avoidance symptoms, while increase in social media use predicted more severe depression symptoms among young adults. Increase in online shopping predicted more severe intrusion and anxiety symptoms. At the same time, PIU during the prolonged stressful period predicted more severe intrusion, avoidance, anxiety and depression symptoms in young adults.

**Table 5:** Increase in specific Internet activities use and problematic Internet use during prolonged stressful period and its association with stress, anxiety, and depression symptoms among young adults—standardized regression paths of the tested model (*N* = 353).

Exogenous variables	Endogenous variables	β	*p*	95% CI	*R*^2^	*p*
Online gaming	→ IES-intrusion	0.23	0.239	−0.05, 0.18	0.097**	0.003
Social media use		0.24	0.168	−0.04, 0.23		
Online shopping		0.44*	0.013	0.04, 0.30		
Problematic Internet use		0.40**	0.008	0.04, 0.27		
Online gaming	→ IES-avoidance	0.38*	0.041	0.01, 0.25	0.068*	0.020
Social media use		0.20	0.211	−0.05, 0.24		
Online shopping		0.09	0.586	−0.10, 0.17		
Problematic Internet use		0.36*	0.011	0.04, 0.28		
Online gaming	→ HADSA	0.18	0.096	−0.02, 0.21	0.171**	<0.001
Social media use		0.17	0.074	−0.01, 0.26		
Online shopping		0.31**	0.001	0.09, 0.35		
Problematic Internet use		0.30**	<0.001	0.11, 0.33		
Online gaming	→ HADSD	0.16	0.105	−0.02, 0.21	0.124**	0.001
Social media use		0.21*	0.017	0.03, 0.31		
Online shopping		0.17	0.054	−0.00, 0.26		
Problematic Internet use		0.19*	0.012	0.04, 0.27		

IES intrusion, Impact of Event Scale intrusion; IES avoidance, Impact of Event Scale avoidance; HADSA, Hospital Anxiety and Depression Scale anxiety; HADSD, Hospital Anxiety and Depression Scale depression.

*p < 0.05; **p < 0.01.

## Discussion

We found increased PIU and problematic specific Internet activities among young adults in the period of prolonged stress due to the pandemic and earthquakes in comparison with the period before the pandemic and earthquakes and in comparison with early and middle-aged adults. We also found that increase in PIU and Internet specific activities use was associated with stress, anxiety, and depression symptoms during the prolonged stress period among young adults.

The current study found a significant increase in overall Internet use (per hour), social media use, and online shopping during the pandemic and earthquakes in comparison with the period before the prolonged stress caused by the pandemic and earthquakes. These results are consistent with previous reports (6, 25, 26). Estimates showed that PIU during the pandemic ranged from 6 to 9.7%, and it was predicted that PIU would increase during the pandemic due to longer time spent on the Internet (6). Although recent studies have indicated an increased prevalence of PIU during the pandemic when compared with the pre-pandemic period, methodologically more rigorous studies should be performed to address psychodiagnostic evaluation and cultural differences and to assess the use of Internet-specific activities (6).

In our study, PIU, problematic social media use, and uncontrolled online shopping increased during the prolonged stress period. The odds for such behavior increased several-fold in comparison with the period before the pandemic and earthquakes. These findings are similar to those already reported for PIU during the pandemic among young adults (39, 59, 60) and after earthquakes among students (56), as well as among adults during pandemic (31, 32), among adults during pandemic and earthquakes (64), and after the Wenchuan earthquake among adolescents (58) or Italian students 2 years after the L’Aquila earthquake (56). Social online networks during natural disasters are important for maintaining interpersonal relationships, communication, education, and work-related obligations. However, excessive use of social networks leads to undesirable consequences, such as increased online activity and development of problematic social media use, including problematic smartphone use in adults (25, 75–77) and young or early adults (78–80). The pandemic-related restrictions led to increased online shopping as the most convenient way to prevent exposure to the virus and remain in a safe environment. In our study, uncontrolled online shopping was equally increased in young adults and older population (48, 64). Uncontrolled shopping can turn into compulsive buying during a prolonged stress, which may be associated with regulation of unpleasant emotional states (49, 64, 81).

A significant increase in gambling were reported for vulnerable sub-populations, such as young adults and persons with prior risky gambling behavior (82). Because only 12 participants in our study reported online gambling, they were excluded from further analysis to avoid a meaningful inferential analysis (83). A scoping review article concluded that the influence of COVID-19 pandemic on gambling behavior and gambling problems is not clear enough and more research is needed on the topic with problem gamblers and vulnerable groups like adolescents and young adults (37).

In our study, there was no increase in online gaming and pornography viewing, although these Internet activities may be particularly relevant during prolonged stress (43, 84). Gaming was recognized as a coping mechanism against stress (85). One study reported increased online gaming during COVID-19 pandemic among Indian college students (41) and another study among Malaysian medical students (42). An online international cross-sectional survey found a minor increase in pornography viewing during the quarantine in younger population and male individuals with higher depression, anxiety, and urgency impulsivity (33).

We found that PIU was more frequent among young adults than older adults, which is in line with a study carried out among Bangladeshi youth and adults (59). There was an increase in problematic social media use and problematic online gaming in our sample of young adults, a finding also reported by other authors (59, 86). These activities were probably even more prevalent during the lockdown and quarantine (87) to help reduce the feelings of isolation, social alienation, and loneliness. Internet use seems to be more problematic for younger users (6, 25, 26, 33, 42). Young individuals (aged 18–25 years) likely use the Internet for educational and/or occupational purposes and leisure pursuits, presumably to compensate for the lack of social contact. They may utilize the Internet to relieve stress or as a coping mechanism to deal with psychological stressors and mood states (such as fear, anxiety, and depression), especially throughout the prolonged stressful period (26, 36, 43, 50–55, 57, 58, 64).

In our study, problematic social media use was more common among the young adults who reported only pandemic-related stress than in those without perceived stress. There were no differences in the Internet use in those who experienced earthquake-related stress and both pandemic and earthquake-related stress. These findings may indicate that different sources of stress can have different effects on Internet behavior (88). One possible explanation is that public health restrictions due to COVID-19 pandemic were most stressful for young people due to the limited social interactions, so the use of social media provided a substitute for relationships and opportunities for communication. Additional studies are required to explain the effect of specific stressors related to the pandemic and earthquakes on recreational and problematic Internet use and specific Internet activities in different stressful situations.

We found that PIU during the prolonged stressful period predicted more severe intrusion, avoidance, anxiety, and depression symptoms in young adults. Other studies have also shown that younger age groups were more vulnerable to stress, depression, and anxiety symptoms (50, 52–58) and that these persons required more support and specific interventions. The standardized regression paths of the tested model showed that increase in online gaming during the prolonged stressful period predicted more severe avoidance symptoms, increase in social media use predicted more severe depression symptoms and increase in online shopping predicted more severe intrusion and anxiety symptoms. The factors potentially mediating the relationship between stress, mental health, and Internet use and specific Internet activities during stressful periods should be further explored and taken into account when undertaking specific measures to mitigate psychological distress and support vulnerable groups (89). Our current findings corroborate the role of depression, anxiety, and stress in PIU and specific Internet activities in response to prolonged stressful experience among young adults.

### Limitations and strengths

The first limitation of our study is inherent to its cross-sectional design, which precludes causal conclusions. Second, the obtained results cannot be extrapolated to the general population due to selection bias. Additionally, recall may be potentially limited because of the retrospective nature of the questionnaire. The survey was not completed by 10% of individuals, which may be connected with the length of the survey (i.e., 15–20 min) and the fact that the respondents did not receive financial compensation.

The survey was based on self-reported data (65). As no standard consensus on diagnostic criteria for PIU and IA is available, and the concept of IA has been criticized (4, 6), we used PIU as a term encompassing gambling, gaming, pornography viewing, excessive social media use, and uncontrolled buying, to screen for and identify at-risk individuals and investigate their specific Internet-based behavior. Still, the self-report bias should be taken into account (89).

Changes in PIU and specific Internet activities were self-reported, as were the assessments of perceived stress in relation to the pandemic and/or earthquakes, which may have led to measurement errors. Furthermore, author-designed questions about pandemic-related stressors may also be the source of possible measurement errors (90). No detailed eligibility criteria, such as absence of neurological conditions or other disorders, or any aspects that affect development; or previous infection or infection in any of the pandemic waves, may be a hindrance. These factors, even if not controlled for or having no confounding effect, should be considered in the light of cross-sectional and online nature of our study design. Still, our results are in line with those reported in other cross-sectional researches in other settings and may have significant impacts for further researches and health-related interventions.

Further prospective research focusing on specific types of stressors (pandemic and earthquake) and their impact on the PIU and specific Internet activities in situations of long-term stress is needed to determine the different effects of various stressors on Internet behavior. Furthermore, it would be of great importance to conduct research studies on clinical samples with age- and culture-appropriate assessment instruments to screen, diagnose, and measure the severity of different forms of Internet-based addictive behaviors. In order to reduce the negative effects of such disasters, the relationship between mental health, associated comorbidities, risk factors, vulnerable groups, quality of life, and Internet-based addictive behaviors should be explored to guide prevention and treatment interventions.

## Conclusion

Our study showed the significant increase in daily overall Internet use and specific Internet activities during prolonged stressful period compared with that before the pandemic and earthquakes. PIU and problematic specific Internet activities were more frequent among young adults than among older participants, and the pandemic-related stress was the most difficult experience for them. More severe intrusion, avoidance, anxiety, and depression symptoms predicted PIU and Internet specific activities in young adults. Depression, anxiety, and stress symptoms played a role in PIU and specific Internet activities during the prolonged stress caused by the COVID-19 pandemic and earthquakes. Differences in the effect of the pandemic and earthquakes on Internet use could reflect the differences in the effect of various stressors on Internet behaviors.

## Data availability statement

The raw data supporting the conclusions of this article will be made available by the authors, without undue reservation.

## Ethics statement

The studies involving human participants were reviewed and approved by the ethics committee of the University Psychiatric Hospital Vrapče in Zagreb, Croatia. The patients/participants provided their written informed consent to participate in this study.

## Author contributions

ZK, TP, and DK-K conceived, designed, and planned the study. MB analyzed and interpreted the data. ZK wrote the first draft. All authors critically revised the content for important intellectual contribution and agreed to the final version of the manuscript.

## Conflict of interest

The authors declare that the research was conducted in the absence of any commercial or financial relationships that could be construed as a potential conflict of interest.

## Publisher’s note

All claims expressed in this article are solely those of the authors and do not necessarily represent those of their affiliated organizations, or those of the publisher, the editors and the reviewers. Any product that may be evaluated in this article, or claim that may be made by its manufacturer, is not guaranteed or endorsed by the publisher.
